# Rotavirus vaccination and the risk of type 1 diabetes and celiac disease: A systematic review and meta-analysis

**DOI:** 10.3389/fped.2022.951127

**Published:** 2022-08-26

**Authors:** Xue Zhang, Xiang-fei Xu, Jie Jin

**Affiliations:** Department of Infectious Diseases, The Affiliated Hangzhou First People’s Hospital, School of Medicine, Zhejiang University, Hangzhou, China

**Keywords:** rotavirus vaccine, celiac, diabetes, systematic review, meta

## Abstract

**Background:**

Rotavirus vaccination has been proven to effectively protect against rotavirus gastroenteritis. However, there are concerns about the relationship between rotavirus vaccination and the risk of autoimmune disorders. Thus, we conducted a systematic review and meta-analysis to comprehensively assess the association between rotavirus vaccination and type 1 diabetes (T1D) or celiac disease (CD) risk.

**Methods:**

A systematic review and meta-analysis were conducted to evaluate the type 1 diabetes or celiac disease associated with rotavirus vaccination. The following journal databases were searched to identify potential studies for inclusion: PubMed, Embase, and Cochrane Library databases.

**Results:**

Seven articles involving more than 5,793,055 children were included. Our results showed that rotavirus vaccination does not alter the subsequent risk of T1D (RR 0.94, 95% CI: 0.82–1.09) or CD (RR 0.86, 95% CI: 0.64–1.17) after vaccination. Furthermore, the risk of T1D was not increased or decreased for children fully exposed to rotavirus vaccination (RR 0.86, 95% CI, 0.54–1.36) and for children partially exposed to rotavirus vaccination (RR 1.05, 95% CI, 0.87–1.26). However, younger (<5 years) vaccinated children at the end of study (RR 0.84, 95% CI = 0.75–0.95) may be at a lower risk for T1D than older (≥5 years) vaccinated children (RR 0.93, 95% CI, 0.81–1.07).

**Conclusion:**

The findings of this study suggest that rotavirus vaccination does not appear to be associated with T1D or CD in children. The protective effect of rotavirus vaccination on T1D may be presented by time dependent.

## Introduction

Rotavirus infection is the most common cause of acute gastroenteritis in children under 5 years of age worldwide (1). In 2009, the World Health Organization recommended that rotavirus vaccines be included in national immunization programs to prevent rotavirus gastroenteritis (2). Since its inclusion in routine immunization programs, gastroenteritis- and diarrhea-associated mortality has markedly decreased in young children, especially infants (3). Several low-income countries have failed to achieve the recommended population coverage for rotavirus vaccination. One possible reason for this failure is concern regarding a possible relationship between vaccination and autoimmune disorders (4).

Rotavirus infection is a risk factor for type 1 diabetes (T1D) (4) and celiac disease (CD) (5). For T1D, viral protein 7(VP7) protein of rotavirus could bind to human leukocyte antigen molecules associated with T1D and elicit T-cell proliferative responses (6). One Australian study observed a potential association between rotavirus infection and increase in antibodies against insulinoma antigen 2 (7). Animal study also demonstrated that rotavirus infection could accelerate the onset of T1D among genetically susceptible mice that spontaneously develop the disease (8). For CD, researcher found that a subset of anti-transglutaminase IgA antibodies also recognize rotavirus VP7, and such antibodies increase intestinal permeability and induce monocyte activation (9). One epidemiologic study (10) showed that that frequent rotavirus infection predicted a higher risk of CD autoimmunity in children with higher risk of CD. However, the incidence of rotavirus infection has declined since introduction of rotavirus vaccination for children in the worldwide. Therefore, it is biologically plausible that a live, attenuated rotavirus vaccine seemed to reduce the risk of T1D or CD in early childhood. Several studies without a control group have reported that the introduction of rotavirus vaccination was followed by a reduction in the annual increase in the incidence of T1D among children aged 0–4 years (11, 12). Epidemiological studies (13–19) of the association between rotavirus vaccination and the risk for T1D have reported inconsistent findings. In a Finnish study, Hemming-Harlo et al. (14) reported that rotavirus vaccination does not increase the risk of T1D, but may decrease the risk of CD. Conversely, a recent study (19) from the United Kingdom did not find evidence for an effect of rotavirus vaccination on the risk of CD or T1D. Therefore, there is no consensus on the relationships between rotavirus vaccination and the risk of T1D and CD. Due to the aforementioned concerns, we performed a systematic review and meta-analysis to quantitatively data from studies undertaken in different countries regarding the associations between rotavirus vaccination and the risk of T1D and CD.

## Methods

### Search strategy

This systematic review and meta-analysis are reported in accordance with the Meta-Analysis for Observational Studies in Epidemiology (MOOSE) checklist (20) (Supplementary Table 1) and Preferred Reporting Items of Systematic Reviews and Meta-analysis (PRISMA) guidelines (Supplementary Table 2). PubMed, Embase, and the Cochrane Library were searched to identify relevant peer-reviewed studies published before July 2021. Synonymous terms were combined to develop the search strategy. The search terms used were “rotavirus vaccination OR rotavirus vaccine” and “diabetes OR celiac OR celiac.” In addition, reference lists of the retrieved articles and relevant reviews were reviewed to identify potential studies possibly meeting the inclusion criteria.

### Study selection

Observational studies were included if they were published as a peer-reviewed article; had a cross-sectional, case-control, or cohort design; compared the risk of T1D or CD between rotavirus vaccine exposure and non-exposure groups; reported incidence rate ratios (IRRs), odds ratios (ORs), relative risks (RRs), or hazard ratios (HRs); and provided adequate data to allow calculation of risk estimates when adjusted data were not provided. Case reports, case series, animal studies, editorials, and reviews were excluded.

### Data extraction and quality assessment

Data were independently extracted by two authors; any discrepancies were resolved through discussion with another author. The extracted data included the first author’s name, publication year, study design, study location, study period, participant characteristics, method of diagnosis of T1D or CD, statistical adjustments, and study quality. In cases where more than one estimate was provided, the most adjusted effect size estimates reported by each study were used. We assessed the methodologic quality of the included observational studies based on the Newcastle-Ottawa Scale (NOS) as recommended by the Cochrane Collaboration (21). The scale assesses studies based on eight criteria, and yields scores ranging from 0 (high risk of bias) to 9 (low risk of bias). Studies with scores > 7 were considered to be of high quality. RCTs were classified as high-quality studies. Summary bias risk assessments were derived for each study.

### Data analysis

All analyses were performed in accordance with the Cochrane Collaboration guidelines using Stata 12.0 meta-analysis software (Stata Corp., College Station, TX, United States). Statistical heterogeneity of the included studies was calculated by the χ^2^ test and I^2^ statistic; an I^2^ of >50% or *p*-value < 0.05 for the Q-statistic was considered to indicate substantial heterogeneity (22). Data were pooled using a random effects model and the generic inverse variance method, as described by DerSimonian and Laird. ORs and 95% confidence intervals (CIs) were calculated for the associations between rotavirus vaccination and subsequent risk of T1D and CD (23). Because of the low absolute risk for T1D or CD in the general population, ORs were considered as approximations of RRs, HRs, and IRRs. Publication bias was evaluated using the Begg funnel plot (24). Publication bias was not formally assessed because each meta-analysis included fewer than 10 studies (25). A *p*-value < 0.05 was considered to indicate statistical significance.

## Results

### Search results

**FIGURE 1:**
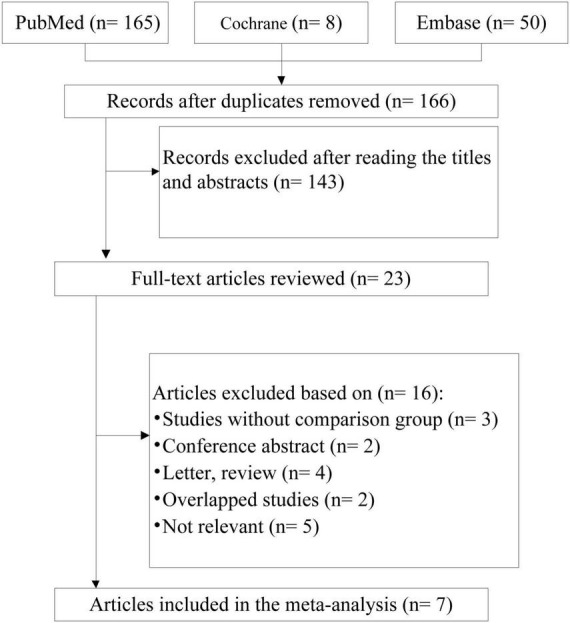
Flow chart of the studies considered and finally selected for review.

After excluding duplicate reports, we identified 166 citations. Based on the titles and abstracts of the papers, 143 were excluded, and the remaining 23 were evaluated on the basis of the full-text articles. Seven studies met the inclusion criteria. Some of the excluded studies, along with reasons for their exclusion, are shown in Figure 1.

### Descriptive characteristics of the included studies

Table 1 summarizes the included studies in detail. This meta-analysis included five cohort studies, one time-series analysis, and one randomized controlled trial published between 2017 and 2021. All included studies were performed in low-mortality settings, with three in Europe, one in Australia, and the other three in United States. Three studies evaluated the associations between rotavirus vaccines and the risk of T1D and CD, and four assessed the association between rotavirus vaccines and the risk of T1D. Four studies evaluated the RotaTeq vaccine (Merck, West Point, PA, United States) and three evaluated the RotaTeq or Rotarix vaccines (GlaxoSmithKline, Rixensart, Belgium). The age range of children at the end of study was varied among the included studies. All studies had high methodological quality (Supplementary Table 3).

**TABLE 1:** Characteristics of the included studies.

Author/coutry	Study design/setting	Born time	End of study	Age at the end of study	Type of vaccine	Vaccine group	No vaccine group	Outcome reported	Diagnostic methods of T1D and CD	Confounding Adjusted (Yes/No)	Quality
Vaarala et al. (13), Finland	Cohort, population-based	2009–2011	2014	3–5 years	RotaTeq	94,437	27,213	T1D; CD	National Care Register	Yes	7
Hemming-Harlo et al. (14), Finland	RCT, population-based	2001–2003	2015	12–14 years	RotaTeq	3,184	2,580	T1D; CD	National Care Register	Yes	8
Perrett et al. (15), Australia	Time-series analysis, population-based	2000–2015	2015	6 months–14 years	RotaTeq	NA	NA	T1D	National Diabetes Services Scheme	No	7
Rogers et al. (16), United States	Cohort, population-based	2001–2017	2017	6 months–11 years	RotaTeq or Rotarix	1,940,963	793,572	T1D	ICD-9 and ICD-10 diagnosis codes	Yes	8
Burke et al. (4), United States	Cohort, population-based	2006–2017	2017	6 months–11 years	RotaTeq or Rotarix	1,245,255	318,285	T1D	ICD-9 and ICD-10 diagnosis codes	Yes	8
Glanz et al. (18), United States	Cohort, population-based	2006–2014	2017	3–11 years	RotaTeq or Rotarix	375,934	111,003	T1D	ICD-9 and ICD-10 diagnosis codes	Yes	8
Inns et al. (19), United Kingdom	Cohort, population-based	2010–2015	2020	7 years	Rotarix	537,516	343,113	T1D; CD	A recorded diagnosis of CD or the prescription of gluten-free goods; A recorded diagnosis of T1D	Yes	8

CD, celiac disease; ICD, international classification of diseases; NA, not available; RCT, randomized controlled trial; T1D, type 1 diabetes.

### Meta-analysis

**FIGURE 2:**
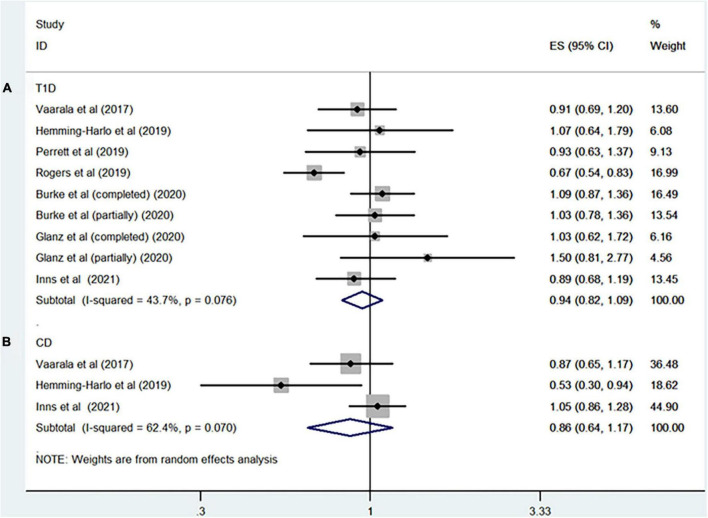
Rotavirus vaccination exposure and the subsequent risk of **(A)** T1D and **(B)** CD.

**FIGURE 3:**
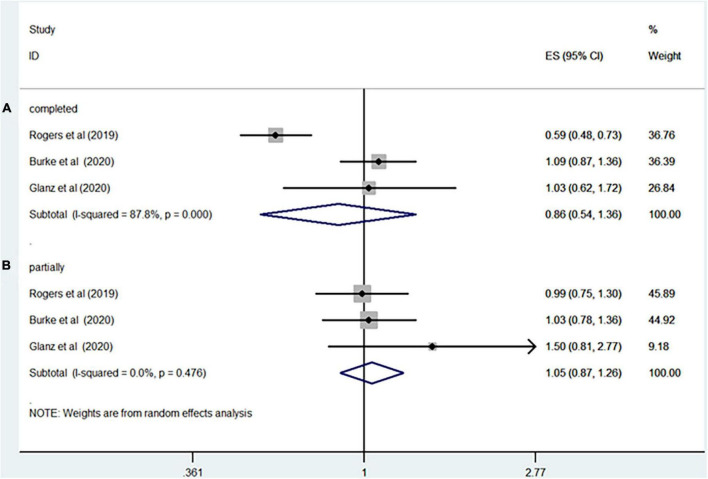
Rotavirus vaccination exposure and the subsequent risk of T1D **(A)** complete vaccination **(B)** partially vaccination.

**FIGURE 4:**
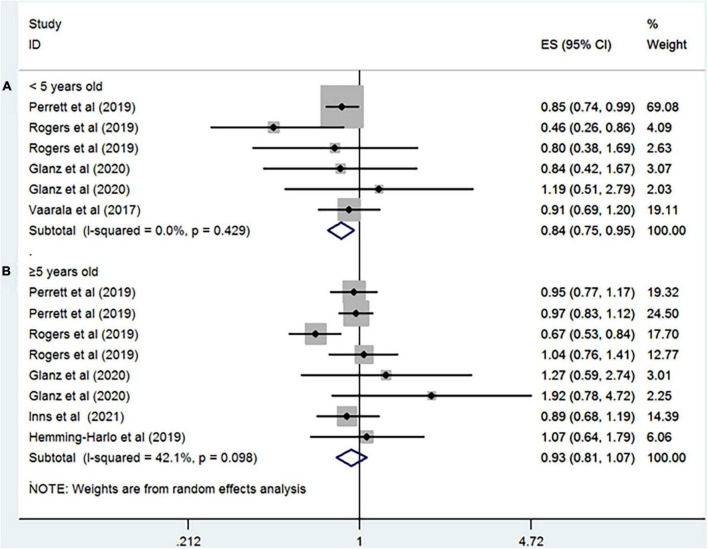
Rotavirus vaccination exposure and the subsequent risk of T1D **(A)** aged < 5 years at the end of study and **(B)** aged ≥ 5 years at the end of study.

Seven studies including more than 5,793,055 participants reported the association between rotavirus vaccine and T1D. The results showed that rotavirus vaccination did not increase the risk of subsequent T1D (RR = 0.94; 95% CI = 0.82–1.09; *p* = 0.41; Figure 2A). Moderate heterogeneity was observed among the studies (I^2^ = 43.7%). Analysis of the cohort studies alone indicated that the combined RR of T1D was 0.95 (95% CI = 0.81–1.11; *p* = 0.5; I^2^ = 50.8%). The RR of T1D was 0.86 (95% CI = 0.54–1.36; *p* = 0.51; I^2^ = 87.8%; Figure 3A) after complete rotavirus vaccination, and 1.05 (95% CI = 0.87–1.26; *p* = 0.62; I^2^ = 0%; Figure 3B) after partial vaccination. Four studies reported the risk of T1D after RotaTeq exposure (RR = 0.98; 95% CI = 0.82–1.18; *p* = 0.852; I^2^ = 0%). In analyses by age at the end of study, vaccinated children aged less than 5 years were at a decreased risk for T1D (RR = 0.84; 95% CI = 0.75–0.95; *p* = 0.006; I^2^ = 0%; Figure 4A), whereas those aged over 5 years were not (RR, 0.93; 95% CI, 0.81–1.07; *P* = 0.299; I^2^ = 42.1%; Figure 4B).

Three studies involving 1,008,043 participants evaluated the relationship between rotavirus vaccination and risk of CD (RR = 0.86; 95% CI = 0.64–1.17; *p* = 0.34; I^2^ = 62.4%; Figure 2B).

## Discussion

To the best of our knowledge, this is the first meta-analysis to evaluate the risk of T1D or CD following rotavirus vaccination. Our results suggested that administration of rotavirus vaccination did not increase the risk of T1D and CD, with pooled RRs of 0.94 (95% CI: 0.82–1.09) and 0.86 (95% CI: 0.64–1.17), respectively. However, a decreased risk of T1D was observed in vaccinated children aged less than 5 years at the end of study.

Rotavirus is the most common viral cause of diarrhea in children (1). A number of studies have evaluated the safety of rotavirus infection in children. Previous epidemiologic studies demonstrated that rotavirus infection may increase the risk of CD or islet cell autoimmunity in children, particularly those with genetic susceptibility to autoimmune disorders (10, 26). Since the introduction of routine rotavirus vaccination worldwide, there has been a significant reduction in the incidence of rotavirus gastroenteritis (27). Therefore, it is reasonable to speculate that exposure to rotavirus vaccine may decrease the risk of T1D and CD. Although a substantial number of clinical trials have assessed the safety of rotavirus vaccines worldwide, evidence for the association between rotavirus vaccination and autoimmune disorders is limited. Two recent reviews (4, 28) have summarized the association between rotavirus vaccination and T1D but addressed the inconsistencies regarding this association. However, these reviews have not provided an overall estimation of the effect of rotavirus vaccination on T1D. In addition, previous systematic review did not evaluate the risk of CD.

T1D and CD have high heritability, estimated to be 70 and 80%, respectively, from twin studies (29, 30). Therefore, studies of the association between rotavirus vaccination and the risk of T1D and CD should take family history of autoimmune disorders into consideration. However, most studies included in this meta-analysis did not adjust for family history of T1D and CD. One included study (18) reported that children with a family history of autoimmune disorders were less likely to receive rotavirus vaccination compared to those without a family history of autoimmune disorders; therefore, it is reasonable to speculate that the strength of the associations in our meta-analysis may have been underestimated. Glanz et al. (18) conducted a sensitivity analysis in children with a family history of T1D, and did not find evidence of an association of rotavirus vaccination with T1D. Environmental exposure during early life plays an important role in the development of autoimmune disorders. Previous studies reported that breastfeeding is associated with a decreased risk of T1D and CD in later life (31). Recent research (18) demonstrated that breastfeeding is positively associated with undervaccination, suggesting that true-negative or -positive associations may be obscured by confounding bias. Unfortunately, the included studies failed to control for breastfeeding; future studies should evaluate the role of early life factors (e.g., breastfeeding) in the risk of T1D and CD among children who have received rotavirus vaccination. Notably, these associations may be modulated by the vaccine dose. If rotavirus vaccination is a protective factor, the risk of T1D and CD in fully vaccinated children should be lower compared to partially vaccinated children. Conversely, if rotavirus vaccination is a risk factor, the risk should be higher in fully compared to partially vaccinated children. Although our subgroup analyses did not identify a significant association, the RR for fully vaccinated children was lower than that for partially vaccinated ones. However, these results should be interpreted with caution due to the limited sample size; further studies are required to clarify this issue.

An unanticipated finding was that vaccination in children aged under 5 years was associated with a decreased risk for T1D, implying that rotavirus vaccination has a time-dependent effect on T1D risk. This may result for two reasons. First, rotavirus vaccine efficacy was proved to wane with time in a recent meta-regression study (32). Therefore, it is reasonable to speculate that at least a portion of children aged over 5 years who received the vaccination during infancy are not protected against rotavirus infection. Second, most cases of rotavirus infection occur in children under 2 years of age, with a peak incidence between 6 and 24 months of age. Thus, the off-target effects are prone to be observed among children aged under 5 years. However, our results relating to the age should be treated with caution due to the limited power of the study.

This systematic review and meta-analysis is the first to provide an overall estimate of the effects of rotavirus vaccination on the subsequent risk of T1D and CD. Our meta-analysis has the advantage of the exclusive inclusion of cohort studies, which are less prone to bias. However, this study had several major limitations. First, there are an unknown number of residual confounders. Further well-designed studies that consider more covariates, such as family history of T1D or CD and breastfeeding, are required to evaluate the association between rotavirus vaccination and the risk of T1D and CD. Second, the duration of follow-up in the included studies was not long enough to determine possible long-term effects. Third, the number of eligible studies and sample size for CD were small, which may have influenced the accuracy of our results. Also, no study reported adjusted estimates by gender, which is an important distinction, as boys and girls are known to have differential risks of CD (33). Future studies are needed before we can have a clear picture of the association between rotavirus vaccination and CD. Fourth, all studies were conducted in Europe or North America; no studies were conducted in Asian or African countries. Therefore, the findings of this meta-analysis cannot be generalized to Asian or African populations. Fifth, only one study (14) intended but failed to explore the risk of other autoimmune diseases due to the rarity of the individual outcomes of interest. Additional studies with larger samples are required to examine the association between rotavirus vaccination and other individual autoimmune disorder.

## Conclusion

In conclusion, the results of this meta-analysis failed to demonstrate a role for rotavirus vaccination in the development of CD in children. However, we could not rule out that the protective role of rotavirus vaccination in T1D development was time dependent and further studies are still needed to verify our findings. Our findings support continued worldwide rotavirus vaccination, reducing the burden of rotavirus morbidity in those populations. As there was a small number of studies included in our review, continued evaluation of this association is warranted, and there is a pressing need for new studies with longer follow-up to further explore the relationships between rotavirus vaccination and the risk of T1D and CD in older children.

## Data availability statement

The original contributions presented in this study are included in the article/Supplementary material, further inquiries can be directed to the corresponding author.

## Author contributions

XZ and JJ conceived the study and revised the manuscript critically for important intellectual content and made substantial contributions to the design, acquisition, analysis and interpretation of data. X-FX participated in the design, selection, and analysis and interpretation of data. All authors read and approved the final manuscript.
