# Supplementary material for: Rotavirus vaccination and the risk of type 1 diabetes and celiac disease: A systematic review and meta-analysis
Source: Front Pediatr. 2022 Aug 26;10:951127. doi: 10.3389/fped.2022.951127 (PMC9459138; doi:10.3389/fped.2022.951127)
Supplement: Supplementary file 1 [file Data_Sheet_1.doc]

**Table S1: MOOSE Checklist**

| **Criteria** | | **Brief description of how the criteria were handled in the meta-analysis** |
| --- | --- | --- |
| **Reporting of background should include** | |  |
| √ | Problem definition | Rotavirus infection has been recognized as a risk factor for type 1 diabetes (T1D) and celiac disease (CD). The precise mechanism of potential risk is unknown, but several biological mechanisms have been proposed to explain a possible relationship between rotavirus infection and the risk of T1D or CD. |
| √ | Hypothesis statement | Rotavirus infection increases in the gut permeability and inflammation leading to the activation of the underlying autoimmunity, or rotavirus antigens could trigger autoimmunity via molecular mimicry. |
| √ | Description of study outcomes | T1D or CD incidence after exposure to rotavirus vaccines |
| √ | Type of exposure or intervention used | Exposure to rotavirus vaccines |
| √ | Type of study designs used | Cohort studies, case-control studies and cross-sectional studies. |
| √ | Study population | Children are exposure to rotavirus vaccines and controls |
| **Reporting of search strategy should include** | |  |
|  | Qualifications of searchers (eg, librarians and investigators) | - |
| √ | Search strategy, including time period included in the synthesis and keywords | PubMed and EmBase databases up to July 2021. |
| √ | Effort to include all available studies, including contact with authors | References of all retrieved articles and recent reviews were reviewed. |
| √ | Databases and registries searched | PubMed and EmBase databases |
| √ | Search software used, name and version, including special features used (eg, explosion) | We did not employ a special search software. |
| √ | Use of hand searching (eg, reference lists of obtained articles) | References of all retrieved articles and recent reviews were reviewed. |
| √ | List of citations located and those excluded, including justification | Details of the literature search process are outlined in the flow chart. |
| √ | Method of addressing articles published in languages other than English | We placed restrictions on English. |
| √ | Method of handling abstracts and unpublished studies | The search process was restricted upon peer-reviewed articles. |
|  | Description of any contact with authors. | - |
| **Reporting of methods should include** | |  |
| √ | Description of relevance or appropriateness of studies assembled for assessing the hypothesis to be tested | The inclusion criteria are presented in the “Search strategy and Study selection” section. |
| √ | Rationale for the selection and coding of data (eg, sound clinical principles or convenience) | Study characteristics were extracted independently by two researchers. The most adjusted estimate was included when a study reported more than one risk estimate. |
| √ | Documentation of how data were classified and coded (eg, multiple raters, blinding, and inrerrater reliability) | Data were independently extracted and analyzed by two investigators and final decision was reached by consensus. |
| √ | Assessment of confounding (eg, comparability of cases and controls in studies where appropriate) | Table 1 presents the adjustment factors for each study. |
| √ | Assessment of study quality, including blinding of quality assessors; stratification or regression on possible predictiors of study results | The quality of each study was assessed by two investigators, using the Newcastle-Ottawa Scale. |
| √ | Assessment of heterogeneity | The *Q*-statistic and *I*-squared (*I*^2^) statistic were used to explore the heterogeneity among studies. |
| √ | Description of statistical methods (eg, complete description of fixed or random effects models, justification of whether the chosen models account for predictors of study results, dose-response models, or cumulative meta-analysis) in sufficient detail to be replicated | Description of methods of meta-analyses, sensitivity or additional analyses, and assessment of publication bias are detailed in the “Statistical analysis” section. |
| √ | Provision of appropriate tables and graphics | One main tables and one supplemental tables are provided. One flow chart and three forest plots appear in the main text. |
| **Reporting of results should include** | |  |
| √ | Graph summarizing individual study estimates and overall estimate | Figures 2-4; FigureS1; “Results” section |
| √ | Table giving descriptive information for each study included | Table 1 |
| √ | Results of sensitivity testing (eg, subgroup analysis) | “Results” section |
| √ | Indication of statistical uncertainty of findings | “Discussion” section; |
| **Reporting of discussion should include** | |  |
| √ | Quantitative assessment of bias (eg, publication bias) | “Results” section and “Discussion” section. |
| √ | Justification for exclusion (eg, exclusion of non-English-language citations) | The details of the exclusion of studies are shown in Flow chart. |
| √ | Assessment of quality of included studies | Studies have been analyzed by the quality. |
| **Reporting of conclusions should include** | |  |
| √ | Consideration of alternative explanations for observed results | We discussed that some included studies didn't make adjustment for other factors or only make adjustment for a few important factors, thus, we cannot exclude chance, residual or unmeasured confounding as alternative explanation for our findings. |
| √ | Generalization of the conclusions (ie, appropriate for the data presented and within the domain of the literature review) | We discussed that some of included studies were based on the number of vaccines or confounding factors they adjusted for. |
| √ | Guidelines for future research | We discussed that more studies are needed to assess the risk of T1D or CD in children exposure to rotavirus vaccines . |
| √ | Disclosure of funding source | The authors received no specific funding for this work. |

| **Table S2: PRISMA Checklist**   | **Section/topic** | **#** | **Checklist item** | **Reported on page #** | | --- | --- | --- | --- | | **TITLE** | | |  | | Title | 1 | Identify the report as a systematic review, meta-analysis, or both. | 1 | | **ABSTRACT** | | |  | | Structured summary | 2 | Provide a structured summary including, as applicable: background; objectives; data sources; study eligibility criteria, participants, and interventions; study appraisal and synthesis methods; results; limitations; conclusions and implications of key findings; systematic review registration number. | 2 | | **INTRODUCTION** | | |  | | Rationale | 3 | Describe the rationale for the review in the context of what is already known. | 3 | | Objectives | 4 | Provide an explicit statement of questions being addressed with reference to participants, interventions, comparisons, outcomes, and study design (PICOS). | 3 | | **METHODS** | | |  | | Protocol and registration | 5 | Indicate if a review protocol exists, if and where it can be accessed (e.g., Web address), and, if available, provide registration information including registration number. | 4 | | Eligibility criteria | 6 | Specify study characteristics (e.g., PICOS, length of follow-up) and report characteristics (e.g., years considered, language, publication status) used as criteria for eligibility, giving rationale. | 4 | | Information sources | 7 | Describe all information sources (e.g., databases with dates of coverage, contact with study authors to identify additional studies) in the search and date last searched. | 4 | | Search | 8 | Present full electronic search strategy for at least one database, including any limits used, such that it could be repeated. | 4 | | Study selection | 9 | State the process for selecting studies (i.e., screening, eligibility, included in systematic review, and, if applicable, included in the meta-analysis). | 4 | | Data collection process | 10 | Describe method of data extraction from reports (e.g., piloted forms, independently, in duplicate) and any processes for obtaining and confirming data from investigators. | 4-5 | | Data items | 11 | List and define all variables for which data were sought (e.g., PICOS, funding sources) and any assumptions and simplifications made. | 4-5 | | Risk of bias in individual studies | 12 | Describe methods used for assessing risk of bias of individual studies (including specification of whether this was done at the study or outcome level), and how this information is to be used in any data synthesis. | 4-5 | | Summary measures | 13 | State the principal summary measures (e.g., risk ratio, difference in means). | 5 | | Synthesis of results | 14 | Describe the methods of handling data and combining results of studies, if done, including measures of consistency (e.g., I^2^) for each meta-analysis. | 5 |   **Table S3**  NOS for Assessment of Quality of Included Studies: Cohort Studies | | | | | | | | |
| --- | --- | --- | --- | --- | --- | --- | --- | --- |
| Study | Selection | | | | Comparability | | Outcomes | |
|  | Representativeness of exposed cohort? | Selection of the nonexposed cohort? | Ascertainment of exposure? | Demonstration that outcome of interest was not represent at the start of the study | Comparability of Cohort* | Assessment of outcome | Was follow-up long enough for outcomes to occur | Adequacy of follow up of cohorts |
| Vaarala et al 2017 | ★ | ★ | ★ | ★ | ★ | ★ | ★ | _ |
| Hemming-Harlo et al 2019 | ★ | ★ | ★ | ★ | ★ | ★ | ★ | ★ |
| Perrett et al 2019 | ★ | ★ | ★ | ★ | _ | ★ | ★ | ★ |
| Rogers et al 2019 | ★ | ★ | ★ | ★ | ★ | ★ | ★ | ★ |
| Burke et al 2020 | ★ | ★ | ★ | ★ | ★ | ★ | ★ | ★ |
| Glanz et al 2020 | ★ | ★ | ★ | ★ | ★ | ★ | ★ | ★ |
| Inns et al 2021 | ★ | ★ | ★ | ★ | ★ | ★ | ★ | ★ |
| Note: A star denotes a score of 1; * A maximum of 2 stars can be allotted in this category | | | | | | | | |
